# Alumina Production from Calcium Aluminate Slags with a Novel HCl-Based Metallurgical Process

**DOI:** 10.1007/s40831-025-01100-8

**Published:** 2025-05-08

**Authors:** Maria Bagani, Amalia Bempelou, Michail Vafeias, Danai Marinos, Anastasia Pilichou, Dimitrios Kotsanis, Dimitrios Sparis, Efthymios Balomenos, Dimitrios Panias

**Affiliations:** 1 School of Mining and Metallurgical Engineering, National Technical University of Athens, 15780 Athens, Greece; 2 METLEN SA-Metallurgy Business Unit, Alumina and Aluminum Plant, St Nikolas, 32003 Paralia Distomou, Greece

**Keywords:** Calcium aluminate slag, Aluminum chloride hexahydrate, Crystallization, HCl gas purging

## Abstract

A novel, HCl-based metallurgical process is investigated aiming at the production of *α*-Al_2_O_3_ from calcium aluminate slags. The process includes the following stages: (a) leaching of the slag with aqueous HCl to dissolve the aluminum content and separate SiO_2_ as a filterable precipitate, (b) HCl_(*g*)_ purging precipitation of the dissolved aluminum in the form of aluminum chloride hexahydrate salt (AlCl_3_∙6H_2_O, ACH), (c) partial removal of dissolved metal impurities from the impure ACH by acetone washing and (d) calcination of the higher purity ACH to produce *α*-Al_2_O_3_. Under optimum leaching conditions, approximately 90% of aluminum is successfully extracted. An ACH purity of 97.5% was achieved after the precipitation and purification process. After calcination of the ACH, *α*-Al_2_O_3_ of 98.5% purity was produced.

**Graphical Abstract:**

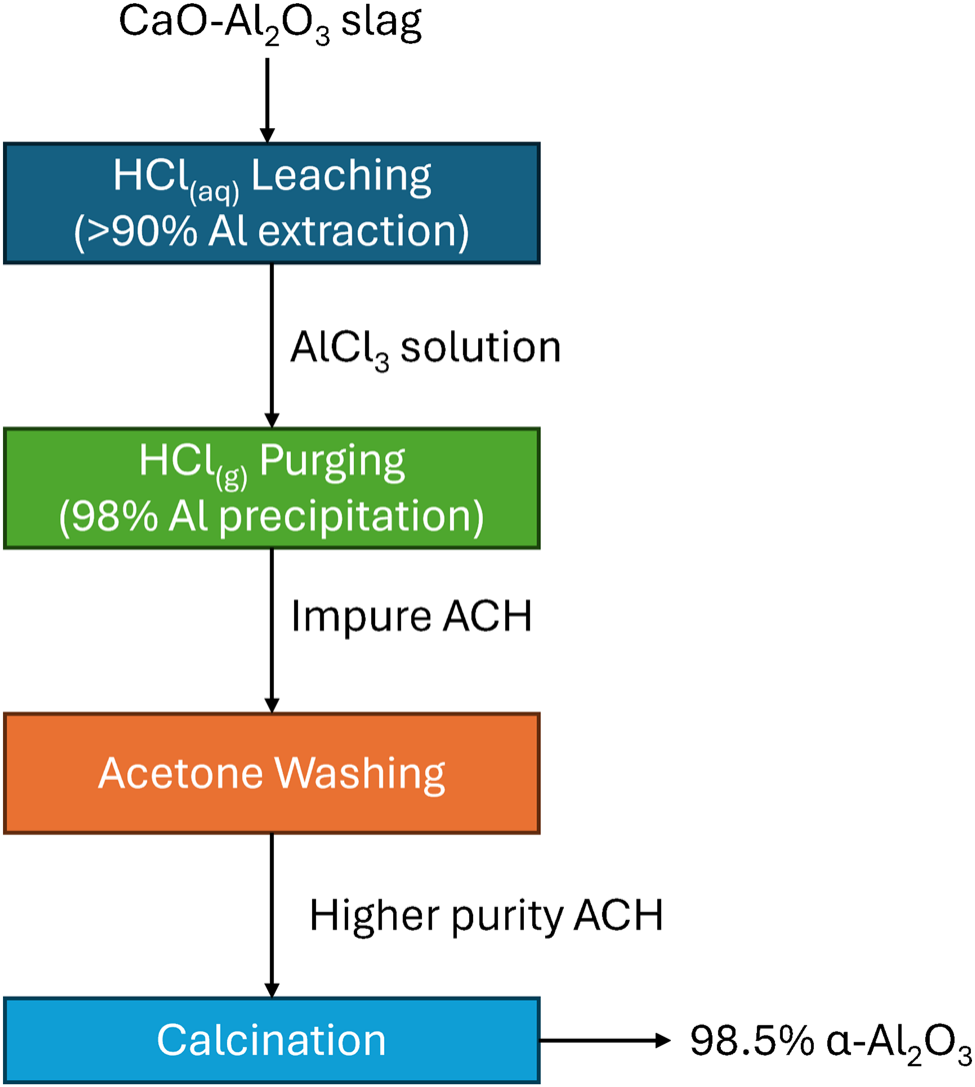

## Introduction

Alumina (Al_2_O_3_) is an important inorganic material with unique physical and technological properties. The global production of Al_2_O_3_ in 2023 is estimated at 400 million tons [1, 2]. Alumina, as a commodity, is divided into two principal grades: (a) smelter grade alumina, suitable for Al metal production, which is produced from the calcination of Al(OH)_3_ by the Bayer process [5] and consists mainly of *γ*-Al_2_O_3_, and (b) specialty-grade alumina, used in other applications including refractories, abrasives, ceramics, pharmaceuticals, pigments, adsorbents [3–5] and have a high *α*-Al_2_O_3_ content [6, 7].Specialty grade aluminas can be further categorized according to their purity level. Among them, High-Purity Alumina (HPA) is a high-purity form of aluminum oxide, corundum (a-Al_2_O_3_). HPA has an Al_2_O_3_ content between 99.99 and 99.999% and is a product of high value and demand, as it is an excellent electrical and thermal insulator [10].

Smelter grade Al_2_O_3_ dominates the market in terms of demand and production rate [8, 9]. However, the alumina obtained from the Bayer process contains impurities like sodium (Na) and iron (Fe), which impose limitations to its utilization in applications where aluminas of higher purity are needed [10] and chemical grade such as HPA are used.

Presently, most of the global production of chemical grade alumina is achieved by the alkoxide process. Although successful, it relies on Al metal as a precursor, tying it to the primary aluminum supply chain and raising concerns about its sustainability [11]. Moreover, the diminishing availability of high-quality bauxite sources and the costly processing of lower-grade ores are likely to affect the overall cost of the conventional HPA production process [12]. If successful, production of HPA from low-cost Al-resources would further decrease production costs for this niche commodity. Consequently, there is a growing interest in exploring pathways to produce HPA from alternative raw materials with innovative approaches. Such approaches generally focus on processing non-bauxitic Al_2_O_3_-bearing raw materials in acidic media.

Mineral acid processes for the extraction of alumina have been researched since the later part of the 19th century [13, 14]. Since then, no systematic review of the progress made in acid processes of alumina production has been published, although it still is an active research area according to the published works [15–31].

The current state and future trends of HPA production have been reviewed by P. Smith and G. Power [12]. One of the process alternatives described is the “aluminum chloride hexahydrate (ACH) process”. Although the aluminum chloride hexahydrate (ACH) process was originally conceptualized for the treatment of aluminosilicate materials, its core features can be utilized to explore HPA production from other secondary sources as well, such as alumina-rich slags. Such an approach is tested in the framework of the SisAl Pilot Project.

**Fig. 1:**
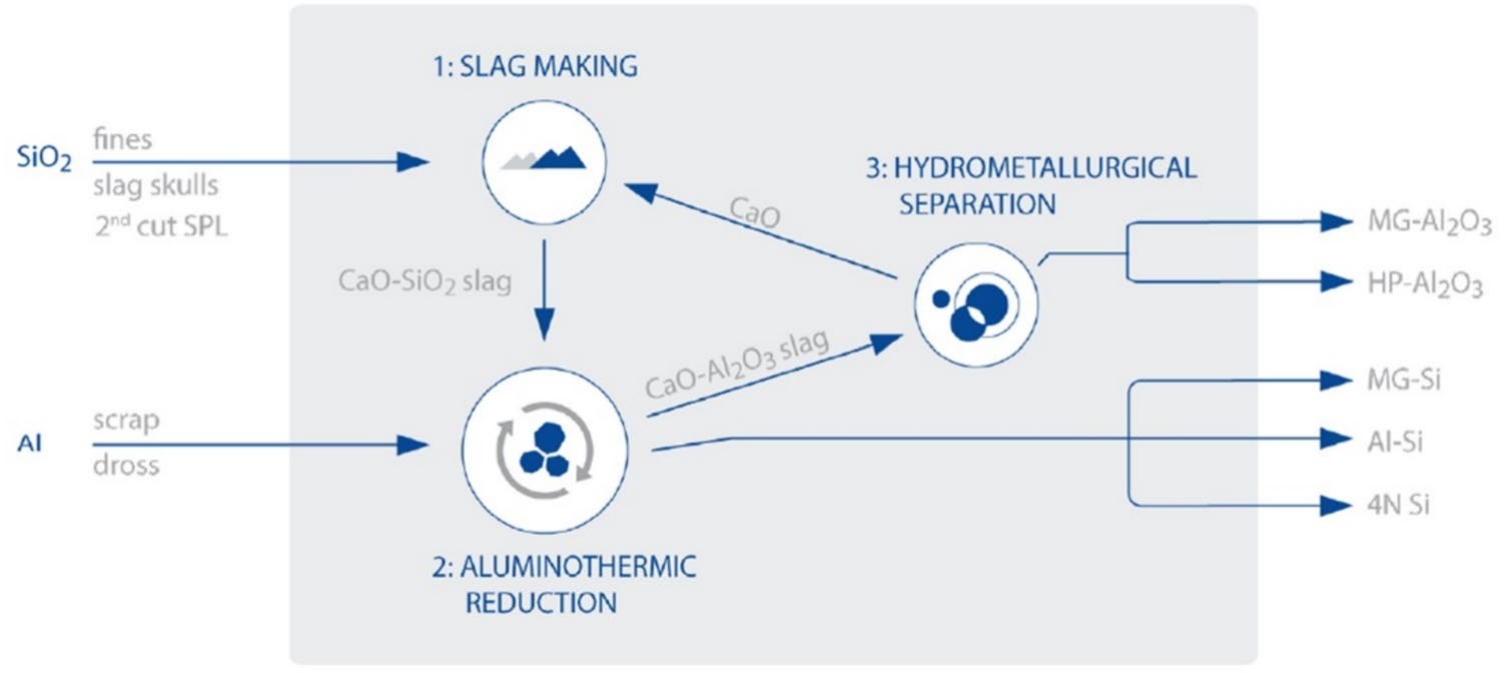
Schematic representation of the SisAl process for the production of different Si and Al_2_O_3_ products [32]

In more detail, the SisAl process aims to demonstrate a sustainable alternative to the energy and emissions intensive submerged arc furnace (SAF) process for Si production [32]. As shown in Fig. 1, the SisAl process produces a liquid CaO-SiO_2_ slag from secondary SiO_2_ sources and then reduces the dissolved SiO_2_ with Al metal (originating from dross, end-of-life scrap, etc.) instead of carbon, thus eliminating direct CO_2_ emissions. The products of this process are metallurgical-grade Si (MG-Si) and calcium aluminate slag (CA slag). The CA slag, due to its high Al_2_O_3_ content and the solubility of AlCl_3_ and CaCl_2_ in HCl solutions, appears as a lucrative raw material for the ACH process. The recovery Al_2_O_3_ in the form of HPA would increase the sustainable and circular character of the process. At the same time, the recovery of CaO is also possible. In more detail, CaO will dissolve in HCl along with Al_2_O_3_, in the form of CaCl_2_(aq). After the separation of AlCl_3_, several strategies can be applied to precipitate CaCl_2_ in a mixture of its hydrate salts, such as evaporative precipitation or anti-solvent precipitation. Afterwards the dehydration and calcination pathways leading to CaO are well documented in literature and have been reviewed by G. Fraissler et al. [35].

**Fig. 2:**
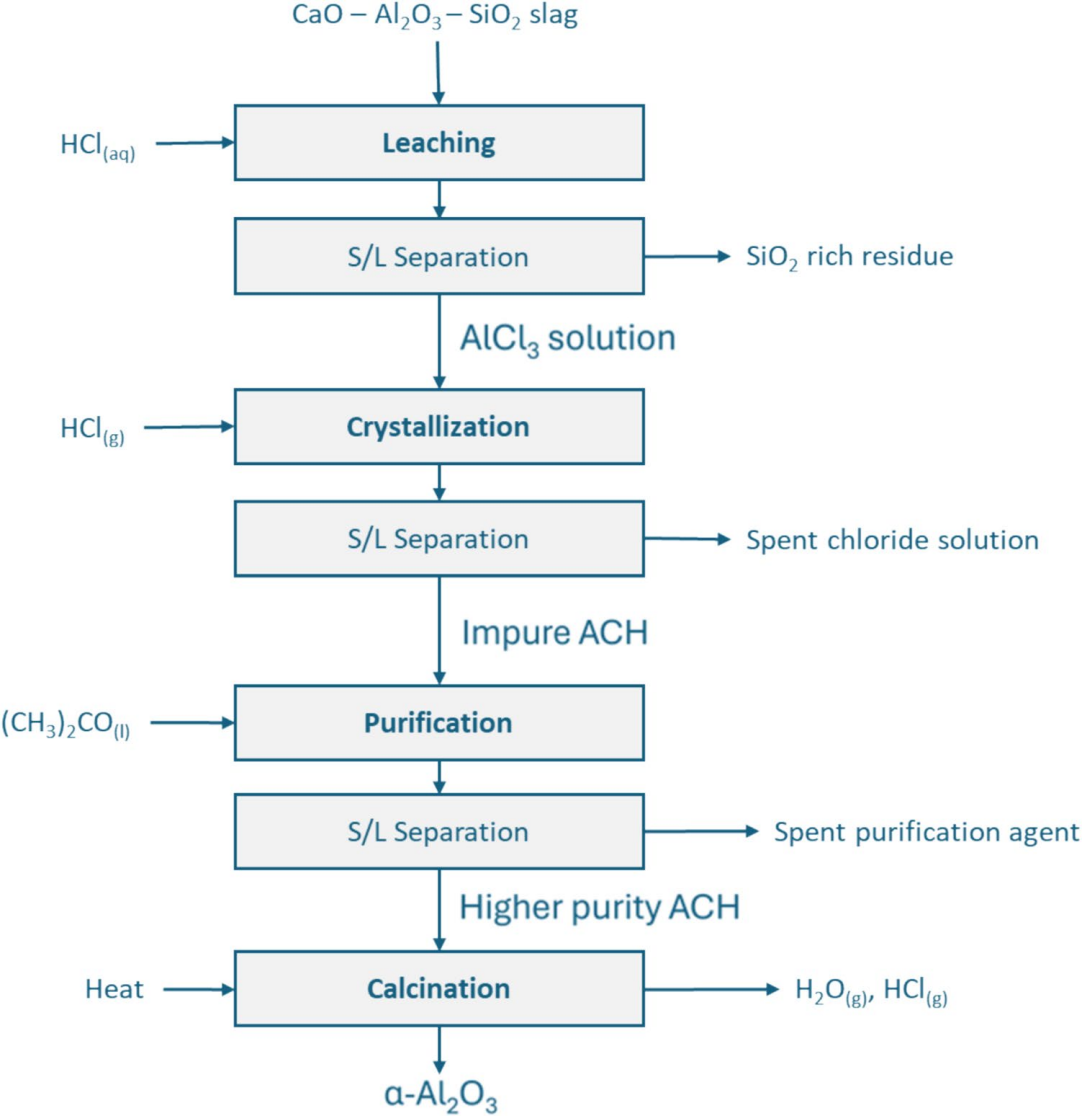
Flowsheet of experimental work conducted in this work

The focus of the present work is the demonstration of a metallurgical process to produce Al_2_O_3_ via an acid route from a CA slag. Not only are all the operations described in detail (leaching of CA slag with aqueous HCl, crystallization of ACH from the PLS, purification of the ACH to remove metal impurities, calcination of ACH to Al_2_O_3_) and presented in Fig. 2, but also detailed data are presented for each operation. Finally, the chemical and mineralogical purity of the Al_2_O_3_ product is presented, thus providing evidence of the potential of our process to be used as a viable, sustainable alternative to produce chemical grade alumina from secondary raw materials. To our knowledge, no other research initiative has presented this potential or shown the interrelations of the numerous metallurgical stages combined to produce Al_2_O_3_ from an aluminate slag. For these reasons the present research is of high value to the fields of sustainable metallurgy, alternative Al_2_O_3_ processes and metal purification processes.

## Materials and Methods

### Leaching of Calcium Aluminate Slag

#### Experimental Methodology

The conditions for optimum leaching have been previously determined with slags produced in laboratory scale furnaces [33, 34] and are not the main focus of the present work. For this study, we replicate the known optimum leaching conditions to the leaching of a CA slag produced in an industrial pilot furnace at ELKEM. The goal of this stage of the work is to produce a pregnant leaching solution (PLS) with a high concentration of Al, while still avoiding silica gel formation. The PLS will be further processed downstream in the crystallization and purification stages.

The slag was leached with 20.2% w/w HCl solution. A simplified chemical equation showing the dissolution of monocalcium aluminate in aqueous HCl is given in Eq. (1).

1 $${\text{CaAl}}_{2}{\text{O}}_{4(\text{s})}+8{\text{HCl}}_{(\text{aq})}\to {\text{CaCl}}_{2(\text{aq})}+{2\text{AlCl}}_{3(\text{aq})}+4{\text{H}}_{2}\text{O}$$

The leaching conditions applied are summarized in Table 1. Leaching tests were repeated three to ensure reproducibility of the results.

**Table 1:** Experimental conditions applied in the leaching stage

S/L Ratio (%)	Temperature (°C)	Agitation (rpm)	Duration (h)
15	80	300	3

The experimental procedure applied is the following: first, the leaching solutions were prepared by the addition of concentrated HCl (37% wt) in deionized H_2_O until the target concentration had been reached. When the leaching solution has reached room temperature, 1.0 L is transferred to the reactor. The reactor is sealed, and mechanical stirring is applied. The solution is preheated to 80 °C. The slag sample is inserted in the reactor when the set temperature is reached and does not fluctuate more than ± 1 °C. The moment the slag is added to the solution is considered the start of each test. After the completion of the test stirring and heating are turned off, the reactor lid is removed and immediately the hot pulp is filtered with a Buchner funnel. After filtration, the resulting chloride solution is left to cool freely to room temperature, its volume is measured and then it is stored in a borosilicate glass vessel. Samples are drawn for chemical analysis from the glass vessels containing the resulting solution. The leaching residue is washed thoroughly with hot deionized Η_2_Ο, placed in a drying oven, and allowed to dry for 24 h at 105 °C before being stored.

#### Laboratory and Analytical Equipment

For the leaching trials, a borosilicate glass reactor was used, equipped with (a) a glass vessel with working volume of 1.0 L, (b) a five-neck borosilicate glass lid, (c) a PTFE shield, and (d) a heating mantle. The openings on the five-neck lid allowed for (a) the insertion of a PTFE-shielded thermocouple for temperature measurements, (b) the insertion of a mechanical stirrer for pulp mixing, (c) the attachment of a condenser for condensing vapors (d) the insertion of the slag sample and (e) the drawing of samples during the leaching. The lid is secured on the reactor body with a clamping ring. The heating mantle, thermocouple, and mechanical stirrer are connected to a PLC unit which allows the control of the operating parameters. Leaching tests were performed in atmospheric pressure conditions.

Elemental chemical analysis of the aqueous solutions produced was performed in a PerkinElmer™ PinAAcle 900 T Atomic Adsorption Spectrometer. ICP-OES elemental analysis was performed in PerkinElmer™ Optima 800 Optical Emission Spectrometer. The mineralogical analysis of the solid samples was conducted using a Rigaku™ Miniflex 600 diffractometer with CuK*α* radiation (*V* = 40 kV and *I* = 15 mA). Phase identification was carried out with Bruker™ Diffrac EVA software, utilizing the ICDD™ PDF-4 + 2023 and PDF-4 Minerals 2023 diffraction databases [36]. Particle size analysis was performed with a Ηοriba Partica LA-960 V2 laser scattering particle size distribution analyzer.

#### Materials Used

Leaching solutions were prepared with concentrated hydrochloric acid (37% HCl, 1.18 g mL^−1^) and distilled H_2_O. Reagent-grade Li_2_B_4_O_7_, LiBO_2_, and KNO_3_ were used for the fusion of the solid samples. Concentrated nitric acid (70% HNO_3_, 1.413 g mL^−1^) and distilled H_2_O were used for the preparation of the aqueous solutions used in dissolving the fused samples for chemical analysis.

Prior to the leaching study, grinding, sampling, and characterization of the slag were performed. In more detail, a representative sample of the CA slag was ground to a particle size − 100 μm and then dried at 105 °C for at least 24 h. Dried samples were fused with a mixture of Li_2_B_4_O_7_/LiBO_2_/KNO_3_ and then dissolved into a 10% HNO_3_ solution. The liquid samples were analyzed by AAS and/or ICP-OES to determine the slag’s composition.

The chemical composition of raw material is shown in Table 2 in equivalent oxide % wt., dry basis.

**Fig. 3:**
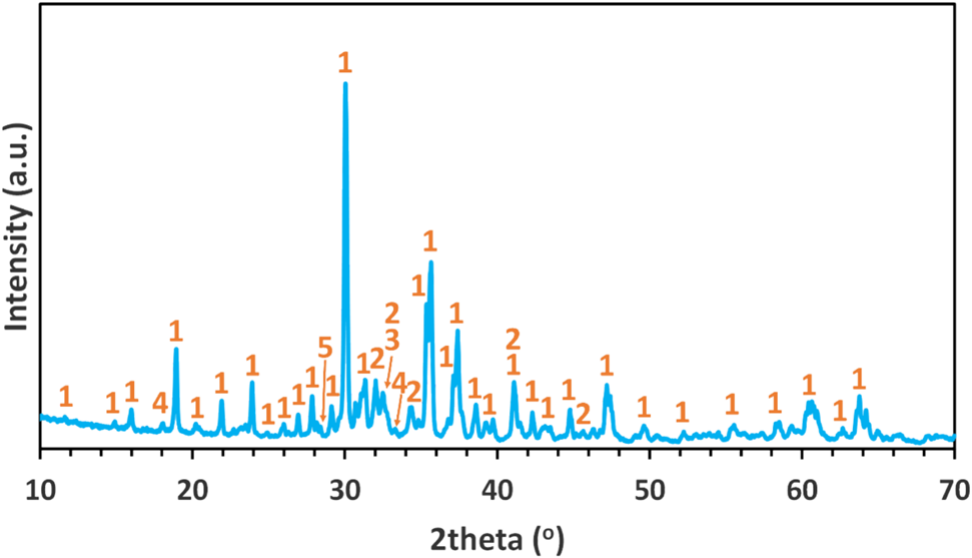
XRD pattern of the slag. Legend: 1-Krotite (CaAl_2_O_4_), 2-Larnite (*β*-Ca_2_SiO_4_), 3-Calcio-olivine (*γ*-Ca_2_SiO_4_), 4-Mayenite (Ca_12_Al_14_O_32.53_), 5-Silicon (Si)

The results of the XRD analysis are presented in Fig. 3.

The slag is predominantly composed of krotite (CaAl_2_O_4_) and larnite (*β*-Ca_2_SiO_4_). Additionally, minor phases such as mayenite (Ca_12_Al_14_O_33_), calcio-olivine (*γ*-Ca_2_SiO_4_), and metallic silicon were identified. The oxidation of the metallic Si during the borate fusion process followed for the chemical analysis is, probably, responsible for the increased equivalent SiO_2_ concentration reported in the chemical analysis of the slag (Table 2), resulting in a sum of equivalent oxides slightly exceeding 100%.

**Table 2:** Chemical analysis of the slag used in this research work

Al_2_O_3_	CaO	SiO_2_	MgO	Na_2_O	Fe_2_O_3_	Total
51.2% ± 1.2%	40.0% ± 2.2%	9.0% ± 0.7%	0.3% ± 0.2%	0.2% ± 0.1%	0.1% ± 0.01%	101.1% ± 1.6%

### Precipitation of ACH

#### Experimental Methodology

The crystallization of ACH by HCl_(g)_ sparging is based on the low AlCl_3(aq)_ solubility in concentrated HCl aqueous solutions. At atmospheric pressure conditions, HCl_(g)_ readily dissolves in aqueous solutions containing AlCl_3(aq)_, as shown in Eq. (2). As a result of the increasing concentration of HCl_(aq)_, aluminum chloride hexahydrate (ACH) precipitates, as shown by the simplified ionic Eq. (3) [37].

2 $${\text{HCl}}_{(\text{g})}\rightleftarrows {\text{HCl}}_{(\text{aq})}$$

3 $${\text{Al}}_{(\text{aq})}^{3+} +{3\text{Cl}}_{(\text{aq})}^{-}+{6\text{H}}_{2}\text{O}\rightleftarrows {\text{AlCl}}_{3}{\bullet 6\text{H}}_{2}{\text{O}}_{(\text{s})}$$

Since most metal chlorides are soluble in aqueous HCl solutions, the major challenge of the HCl sparging crystallization process is the control of metal precipitation and precipitate purity. Several studies have been conducted in the field of impurity removal. Studies by the US Bureau of Mines focused on removing Fe from AlCl_3_ solutions via solvent extraction, along with controlling Mg and P [38–40]. Furthermore, Bremmer [41] proposed: (a) Addition of purification cycles until the desired purity of ACH is achieved [42], where precipitate is re-dissolved in an aqueous solution and, subsequently, HCl sparging precipitation occurs. (b) Washing of the produced ACH with an organic agent, instead of HCl washing. The latter approach seems the most appealing in terms of cost-effectiveness and ease of application. Several organic agents were considered, among them, acetone is attractive, due to its high volatility and its potential to dissolve metal chloride impurities selectively, apart from AlCl_3_.6H_2_O [43]. According to the information provided above, an experimental design was prepared to aim for the following outcomes: (i) maximum ACH precipitation degree, (ii) minimum co-precipitation degree of impurities, and (iii) minimum HCl_(g)_ gas consumption. The experimental work is divided into four stages.

##### Precipitation Stage

HCl_(g)_ was sparged in the AlCl_3_ solution until it was saturated with HCl_(aq)_ (37% w/w). Stirring was applied to aid the dispersion and dissolution of HCl_(g)_ in the aqueous solution. Samples were extracted at predefined intervals. These intervals were defined according to the molar amount of HCl_(g)_ sparged into the solution. The resulting solution was separated from the intermediate ACH precipitate via vacuum filtration. The experimental conditions applied in the precipitation trials are summarized in Table 3. These trials were repeated five (5) times to ensure the reproducibility of the results.

**Table 3:** Experimental conditions applied in the precipitation stage

HCl_(g)_ flow	Temperature (°C)	Agitation (rpm)	Sampling (M of HCl)
Constant, 600 mL/min	20	300	3, 6, 7, 8, 9, 10

##### Washing of Precipitate

The ACH precipitate was washed with puriss p.a. acetone (Honeywell > 99.5%) at a 160 mL/100 g L/S ratio. Washing was performed at room temperature. The ACH/Acetone slurry was then filtered, and two phases were obtained: the impurity-bearing acetone solution and the purified ACH product.

##### Determination of Impurities in Acetone Solution

The impurity-bearing acetone solution was mixed with deionized H_2_O and then boiled to complete evaporation. The solid residue after the evaporation, containing salts of the impurities was dissolved in deionized H_2_O. The samples were analyzed to determine the efficiency of the washing process. TOC (Total Organic Carbon) analysis, performed in the final solution, confirmed the successful and complete acetone remove.

##### Dissolution of Purified ACH for Determination of Chemical Purity

The purified ACH product is dried at 40 °C to remove, by evaporation, any residual entrapped acetone. Then it is dissolved in deionized water to create a pure AlCl_3_ solution. The produced solution is chemically analyzed.

#### Laboratory and Analytical Equipment

For the HCl_(g)_ sparging crystallization experiments, an Amar G2360 glass reactor was used, equipped with a 1.0 L capacity jacketed glass vessel and a polytetrafluoroethylene (PTFE) lid. The lid is designed with openings that allow (a) the immersion of a thermocouple and a 6-bladed turbine PTFE stirrer, (b) the sampling, and (c) the gas inlet and outlet. The mechanical stirrer is connected to a controller while the heating/cooling is controlled by a chiller. The gas outlet socket is immersed in a NaOH solution to neutralize any unreacted HCl. HCl_(g)_ constant flow was achieved by a gas mass flow controller (Bronkhorst series).

The mineralogical and particle size distribution analyses were conducted using the equipment described in “Laboratory & Analytical Equipment” section. To determine the theoretical HCl_(g)_ needed to reach the saturation point, the free HCl content of the PLS had to be determined. Hydrolyzable ions included in PLS (Al^+3^, Fe^+3^_,_ etc.), are masked with EDTA (Chembiotin) [12], and then the solution is titrated with standard NaOH solution (Honeywell) in an auto titrator Model 702 SM manufactured by Metrohm.

### Calcination of ACH to Al_2_O_3_

#### Experimental Methodology

ACH decomposition occurs at elevated temperatures, according to the simplified reaction represented by Eq. (4).

4 $${\text{AlCl}}_{3}{\bullet 6\text{H}}_{2}{\text{O}}_{(\text{s})}\to \frac{1}{2}{\text{Al}}_{2}{\text{O}}_{3(\text{s})}+3{\text{HCl}}_{(\text{g})}+4.5 {\text{H}}_{2}{\text{O}}_{(\text{g})}$$

The formation of alumina from ACH is a complex process, influenced by various factors, with temperature being the most significant. The *α*-Al_2_O_3_ phase is the thermodynamically stable form [44], while other metastable phases exist. For the ACH calcination process, a temperature of 1100 °C is suggested for the formation of the desired phase [18]. The calcination process was applied in a two-step procedure for the collection of HCl fumes produced during calcination at lower temperatures of 150–200 °C [45]. Experimental conditions followed are presented in Table 4.

**Table 4:** Applied conditions on calcination steps

Calcination step	Retention time (h)	Temperature (°C)
1st	1	400
2nd	1	1200

#### Laboratory and Analytical Equipment

The first calcination step was performed in a tube furnace. N_2_ gas was inserted at a constant flow (200 cm^3^/min) controlled by a gas mass flow controller [Bronkhorst series]. Off-gases were driven to a 4 M NaOH solution, to neutralize HCl fumes generated according to Eq. (4). The second step was performed at a laboratory muffle furnace, under open-air conditions. Wet chemical analysis techniques determined the chemical composition of the main metals contained. Samples were fused with a mixture of 1:1 Li_2_B_4_O_7_/LiBO_2_ with the addition of 0.1 g KNO_3_ and then dissolved into a 10% HNO_3_ solution. Liquid samples chemical analyses, and crystallographic and particle size distribution analyses were also performed in the corresponding equipment described in “Laboratory & Analytical Equipment”section.

## Results and Discussion

### Slag Leaching Results

The results of the slag leaching runs are presented in Table 5. In more detail the average wt% extraction for each of the main metals (Al, Ca, Si, Fe, Mg, and Na), along with their corresponding concentration in the PLS are presented.

**Table 5:** % wt. Extraction of major metals in the PLS and their corresponding concentration values

Metal	Al		Ca		Si	
Run	wt% extraction	Concentration (g/L)	wt% extraction	Concentration (g/L)	wt% extraction	Concentration (g/L)
1	93.2%	42.1	89.6%	42.5	3.1%	0.22
2	90.4%	39.3	93.2%	42.6	6.5%	0.48
3	92.2%	38.1	96.6%	42.3	4.1%	0.27
Average	91.9%	40.7	93.2%	42.6	4.6%	0.35
St. dev	± 1.4%	± 2.1	± 3.5%	± 0.2	± 1.7%	± 0.14

Metal	Fe		Mg		Na	
Run	wt% extraction	Concentration (g/L)	wt% extraction	Concentration (g/L)	wt% extraction	Concentration (g/L)
1	61.7%	0.07	83.7%	0.25	1.9%	0.005
2	58.9%	0.07	86.8%	0.25	2.3%	0.006
3	71.9%	0.08	87.1%	0.24	2.5%	0.006
Average	64.2%	0.07	85.9%	0.25	2.1%	0.005
St. dev	± 6.8%	± 0.005	± 1.9%	± 0.006	± 0.3%	± 0.001

The results of Table 5 reveal that the acid leaching of the CA slag is an efficient process. The metal of interest, i.e., Al, has a %wt. extraction value of approx. 92%. The second major component of the slag, i.e., Ca, is almost completely extracted as well, with a %wt. extraction value of 93.2%. A second objective of the leaching process is to avoid silica gelation phenomena, which is also achieved. Almost all the Si remains in the leaching residue and only approx. 350 mg/L Si are dissolved in the PLS. Concerning the other impurities, Mg exhibits the same behavior as Ca and is almost completely dissolved in the PLS. Nonetheless, its concentration in the PLS is approx. 250 mg/L, due to its low concentration in the slag. Fe and Na exhibit the lowest concentrations in the PLS.

Overall, the PLS produced is an AlCl_3_/CaCl_2_ solution with Mg and Si as the major impurities (both > 100 mg/L) and Fe and Na as minor impurities. The solutions produced from the three leaching runs were mixed to form a single batch of PLS that would be used in the ACH precipitation experiments. The batch of PLS was then transferred to a borosilicate glass container, sealed, and stored until the ACH precipitation trials.

### Precipitation of ACH

#### Precipitation Stage Results and Characterization of ACH

**Fig. 4:**
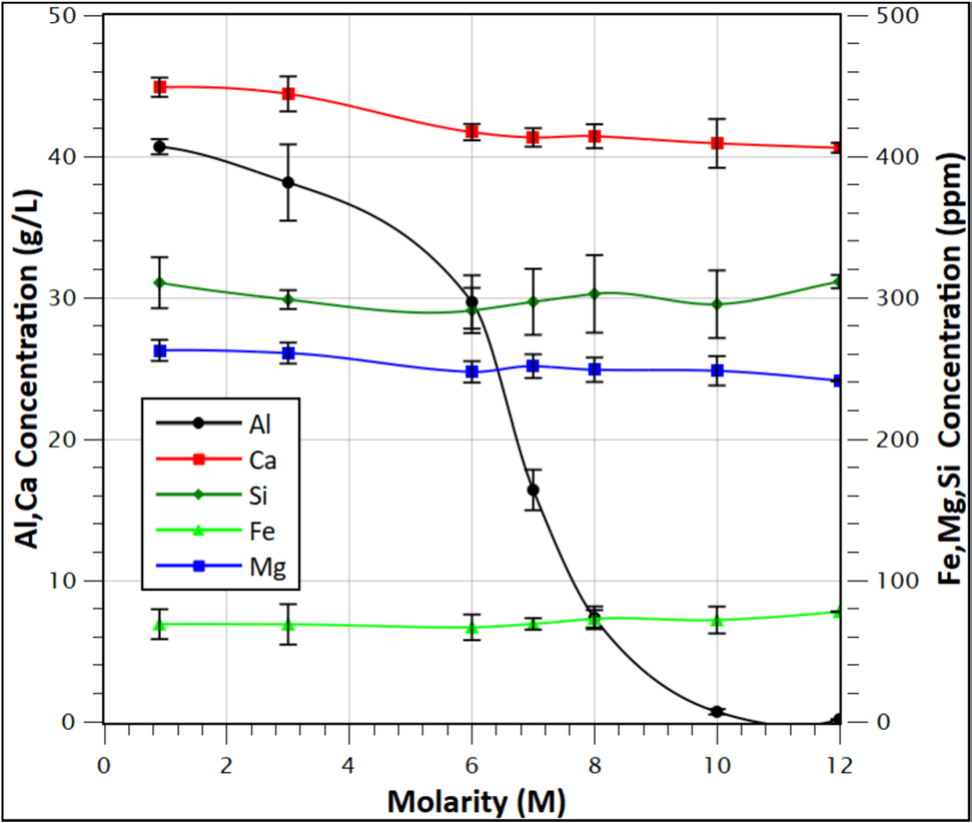
Effect of HCl gas insert on the Al, Ca, Fe, Mg, and Si concentration, (Conditions applied: 300 rpm stirring rate, 600 mL/min HCl flow rate, 20 °C temperature)

The PLS, of the average composition shown in Table 5, underwent HCl_(g)_ sparging crystallization in batch trials. Fig. 4 depicts the average concentration of the dissolved Al, Ca, Fe, Mg, and Si as a function of the final reached HCl molarity. The concentration curves are based on the average values of the five batch trials.

As shown by the Al concentration curve, the concentration in the PLS decreases almost linearly during the first stages of HCl_(g)_ sparging, until approximately 5 M of HCl concentration in the PLS. A steep decrease in the 5 to 8.5 M HCl molarity range is observed, which represents the rapid precipitation area. Considering the impurities (Ca, Mg, Fe, and Si), their concentration remains relatively stable until 10 M HCl molarity, except for Ca. More specifically, until 6 M approximately 7% of Ca is co-precipitated while between 8 and 12 M HCl molarity, 1.8% of Ca coprecipitates. Ca is expected to be precipitated in this range in the form of antarcticite (CaCl_2_·6H_2_O) [46, 47]. Mg shows a small precipitation degree which cannot be accurately calculated and can be partially attributed to the standard analytical error of measurements. Precipitation of Mg in the form of Bischofite (MgCl_2_·6H_2_O) in high HCl molarity is predicted in the work of both Christov [48] and Khoo [49] in the ternary MgCl_2_-H_2_O-HCl system. In the case of Si and Fe, a small precipitation degree is noted which cannot be calculated accurately. According to the literature review, iron is expected to precipitate mainly in the form of FeCl_3_·6H_2_O [50]. Interestingly, the paper suggests that its precipitation is minor in temperatures under 36 °C, which follows our work. Silicon co-precipitation should not occur in the applied conditions, particularly in the low pH values reached during the trials, according to data provided by Carroll [51] and Hartman [52]. However, the irregular shape of both Si and Fe curves can be attributed to their low concentration near the detection limit of the analytical equipment used. As a result of the above observations, 10 M HCl concentration is selected as the optimum, combining a high Al precipitation degree with relatively low co-precipitation of impurities. The precipitation yields at 10 M HCl in solution are provided in Table 6.

**Table 6:** Precipitation yield of elements from the starting PLS at 10 M HCl molarity

Species	Al	Ca	Fe	Mg	Si
Precipitation yield %	98.0 ± 0.54	7.8 ± 0.43	1.9 ± 0.09	4.1 ± 0.12	3.5 ± 0.17

#### Acetone Washing, Removal of Impurities, and Analysis of Purified ACH

The chemical analysis of the produced precipitate after the acetone washing step is presented in Table 7. The ACH composition on a dry basis was not possible to be determined, since drying was performed in a low-temperature furnace (40 °C). Thus, the chemical analysis of the ACH is presented in Table 7, including any remaining humidity.

**Table 7:** Average composition of purified precipitate after acetone washing

Species	Al	Ca	Mg	Si	Cr	K	Na	Fe
Mass %	10.9 ± 0.09	0.2 ± 0.04	0.003 ± 0.0005	0.002 ± 0.0004	0.001 ± 0.0004	0.0004 ± 0.0001	0.003 ± 0.0008	B.D.L

According to the bulk analysis of the purified precipitate, Al purity is found to be 10.9%, equivalent to a 98% ACH purity. In comparison, the co-precipitated impurities are all found under 1%. An interesting remark in the composition of purified ACH, depicted in Table 7, is the absence of Fe content. According to the iron precipitation yield of 1.9% (Table 7), iron was expected to be present in the purified ACH. Its absence shows that Fe content from intermediate ACH was dissolved by acetone washings and passed to the impurities-bearing solution as presented in Table 8.

**Table 8:** Average bulk composition of impurities-bearing solution

Metal	Al	Ca	Mg	Si	Fe
Concentration (g/L)	0.35 ± 0.08	3.22 ± 0.4	0.0009 ± 0.0003	0.008 ± 0.002	0.002 ± 0.0009

This observation agrees with literature where Fe chlorides, such as FeCl_3_*6H_2_O are reported as soluble in acetone [53].On the other hand, Ca and Mg chlorides are reported as slightly soluble in acetone [54]. The presence of additional impurities in the impurities-bearing solution suggests that the washing step increased the purity of ACH by transferring some of the initially co-precipitated impurities to the impurities-bearing solution. While the Al% dissolution yield in acetone, from the intermediate ACH, is low as was expected [55]. As described in 2.2.2 acetone was evaporated from the solution by heating. Acetone could be regenerated from an aqueous solution through distillation [56]. Implementing this step could allow acetone to be reused in the process, enhancing its sustainability.

#### ACH Calcination to Al_2_O_3_

**Fig. 5:**
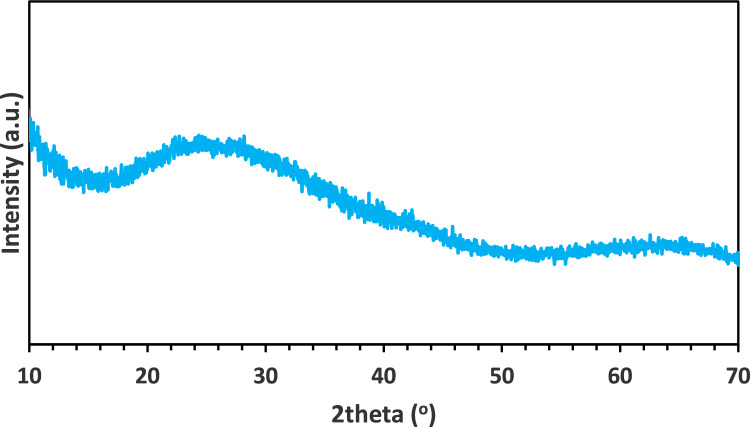
XRD pattern of the calcined ACH from the 1st calcination step

**Fig. 6:**
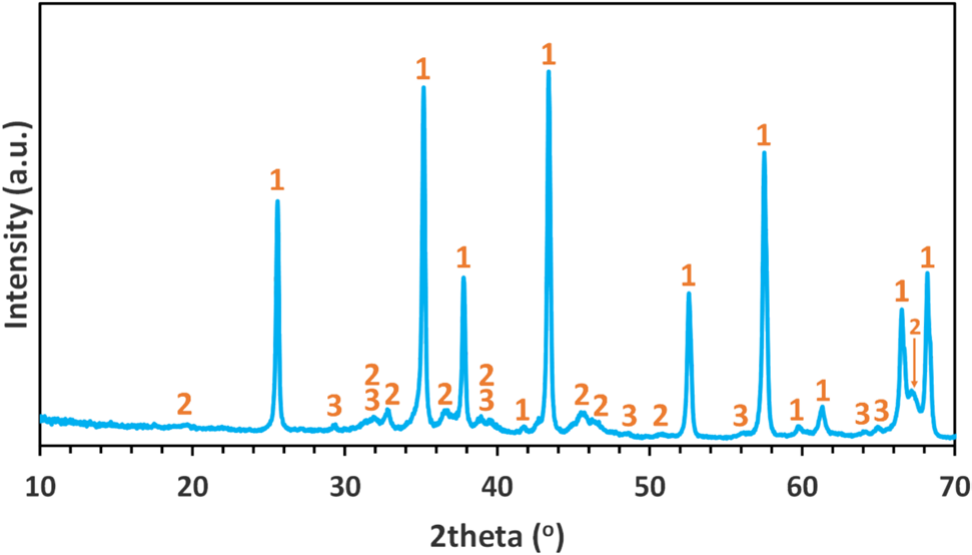
XRD pattern of calcined ACH from the 2nd calcination step. Legend: 1- *α*-Al_2_O_3_, 2- *δ*-Al_2_O_3_, and 3- *κ*-Al_2_O_3_

The qualitative XRD analysis of the products from the 1st and 2nd calcination steps is presented in Figs. 5 and 6, respectively.

The outcome from the 1st calcination step was the formation of an amorphous material, as can be observed from the broad bumps in the XRD pattern, while the product from the 2nd calcination step is composed mainly of corundum (*α*-Al_2_O_3_) and two transitional aluminas in lesser amounts, namely the delta (*δ*-Al_2_O_3_) and kappa (*κ*-Al_2_O_3_) phase. Perander [57] presented data indicating that the thermal decomposition of ACH results in the formation of amorphous Al_2_O_3_, specifically *ρ*-Al_2_O_3_. According to Levin [44], *ρ*-Al_2_O_3_ is the only amorphous metastable crystal structure of Al_2_O_3_. According to the literature on ACH decomposition [57, 58], the thermodynamically stable phase at 1200 °C, as employed in this study, is *α*-Al_2_O_3_. However, the accurate temperature required for the transformation to *α*-Al_2_O_3_ primarily depends on the precursor phase and other parameters, e.g., the duration of calcination, the effect of anionic and cationic impurities, etc. XRD analysis confirmed that the *α* phase is predominant while detecting smaller quantities of the *δ* and *κ* phases. The presence of these metastable alumina phases may be due to kinetic factors or the presence of impurities [59].

Chemical analysis of the final calcined Al_2_O_3_ is presented in Table 9.

**Table 9:** Chemical analysis of produced alumina

Species	A_2_O_3_	Ca	Mg	K	Na	Cr	others
Mass %	98.5 ± 0.3	1.03 ± 0.9	0.02 ± 0.008	0.1 ± 0.03	0.05 ± 0.009	0.08 ± 0.01	0.22

According to the universal standard system [60], the final product is classified as 1N purity Al_2_O_3_. As for the included impurities, calcium was the only one detected over 1% while all the other elements, were found at or below 0.1%.

The produced Al_2_O_3_ consists mainly of the corundum phase and has a purity of over 98%. This material could present a possible feed for numerous applications. Among them, one possible is the ceramic industry and particularly the use of structural parts. The existence of the *α*-Al_2_O_3_ phase provides good mechanical strength, good chemical resistance, and high temperature resistance [61]. However, the existence of intermediate phase and impurities content does not permit its use in more delicate uses such as specific uses ceramics [62], LEDs and Li-Ion battery separators [12]. Application of purification processes, prior to calcination, could increase the Al_2_O_3_ purity and lead to the avoidance of metastable phase formation during calcination enabling the widening of possible applications.

## Conclusion

In this work, an acid route was presented for the production of Al_2_O_3_ from a calcium aluminate slag. The slag is a by-product of the novel SisAl process that utilizes secondary raw materials of the Si and Al sector to produce, via an aluminothermic reduction process, MG-Si. The slag was chemically and mineralogically characterized and then leached in a single stage with a 20.2% w/w HCl solution, under conditions that had been previously determined and are listed in Table 1. Higher than 90% of the Al_2_O_3_ and CaO contents of the slag were dissolved, producing an AlCl_3_/CaCl_2_ PLS containing 40.7 g/L Al and 42.6 g/L Ca, 350 ppm Si, 70 ppm Fe, 250 ppm Mg and 5 ppm Na.

The PLS was then subjected to HCl_(g)_ purging which led to the precipitation of Al in the form of ACH. The yield of Al crystallization till a final 10 M HCl concentration in the PLS was very high (98%) followed by an appreciable crystallization of Ca (almost 8.5%), lower crystallization of Mg (4.1%) and almost negligible crystallization of Si (0.2%) and Fe (0.1%). Analysis of the produced precipitate showed an ACH purity of 97.5%. The obtained AlCl_3_*6H_2_O precipitate after drying at 40 °C and acetone washing contained 0.3% Ca while the rest of the elements were found near or under 0.01%. Acetone washing proved to be very efficient in removing iron impurities from ACH. Calcination of the produced ACH, in a two-step route led to the production of mainly a-Al_2_O_3_, where traces of *δ*-Al_2_O_3,_ and *κ*-Al_2_O_3_ were also detected. The produced Al_2_O_3_ could be used in ceramic applications while implementing purification processes before calcination could lead to a higher purity product with further possible applications and is left for future investigation.
